# Language-Guided Semantic Clustering for Remote Sensing Change Detection

**DOI:** 10.3390/s24247887

**Published:** 2024-12-10

**Authors:** Shenglong Hu, Yiting Bian, Bin Chen, Huihui Song, Kaihua Zhang

**Affiliations:** B-DAT and CICAEET, Nanjing University of Information Science and Technology, Nanjing 210044, China; hslnuist@163.com (S.H.); 202312490781@nuist.edu.cn (Y.B.); 202312490782@nuist.edu.cn (B.C.); zhkhua@gmail.com (K.Z.)

**Keywords:** remote sensing change detection (RSCD), contrastive language-image pretraining (CLIP), clustering, semantic information

## Abstract

Existing learning-based remote sensing change detection (RSCD) commonly uses semantic-agnostic binary masks as supervision, which hinders their ability to distinguish between different semantic types of changes, resulting in a noisy change mask prediction. To address this issue, this paper presents a Language-guided semantic clustering framework that can effectively transfer the rich semantic information from the contrastive language-image pretraining (CLIP) model for RSCD, dubbed LSC-CD. The LSC-CD considers the strong zero-shot generalization of the CLIP, which makes it easy to transfer the semantic knowledge from the CLIP into the CD model under semantic-agnostic binary mask supervision. Specifically, the LSC-CD first constructs a category text-prior memory bank based on the dataset statistics and then leverages the CLIP to transform the text in the memory bank into the corresponding semantic embeddings. Afterward, a CLIP adapter module (CAM) is designed to fine-tune the semantic embeddings to align with the change region embeddings from the input bi-temporal images. Next, a semantic clustering module (SCM) is designed to cluster the change region embeddings around the semantic embeddings, yielding the compact change embeddings that are robust to noisy backgrounds. Finally, a lightweight decoder is designed to decode the compact change embeddings, yielding an accurate change mask prediction. Experimental results on three public benchmarks including LEVIR-CD, WHU-CD, and SYSU-CD demonstrate that the proposed LSC-CD achieves state-of-the-art performance in terms of all evaluated metrics.

## 1. Introduction

Remote sensing change detection (RSCD) involves taking a pair of bi-temporal images as input that differ in time but share the same spatial location, aiming to identify the regions that have changed between the bi-temporal images. RSCD has been widely applied in various surface monitoring scenarios such as disaster assessment [1,2,3], urban planning [4,5,6], and agricultural management [7,8,9].

The traditional methods in RSCD [10,11,12,13,14,15,16,17] identify changed regions in an unsupervised manner by analyzing handcrafted features. However, due to the limited discriminability of handcrafted features, traditional methods struggle to handle increasingly complex RSCD scenarios. Afterward, with the rapid development of deep learning (DL), the data-driven DL paradigm has demonstrated superior performance in learning discriminative feature representations, breaking the representative limitations of the handcrafted features, thereby attracting much attention in RSCD. Daudt et al. [18] introduced a Siamese architecture, consisting of two parallel subnetworks for RSCD. These subnetworks simultaneously extract the features from bi-temporal images and directly analyze changed features. Inspired by this structure, numerous change detection models have emerged that can be divided into two major categories: the convolutional neural network (CNN)-based and the transformer-based methods. The CNN-based methods [18,19,20,21,22,23,24,25,26] aim to enhance local feature discrimination capabilities by using the convolutional architecture [27]. In contrast, to reduce the inductive bias of the CNN architecture, the transformer-based methods [28,29,30,31,32,33,34] focus on improving the long-range modeling abilities via introducing the self-attention mechanism into transformer architectures [35].

**Figure 1:**
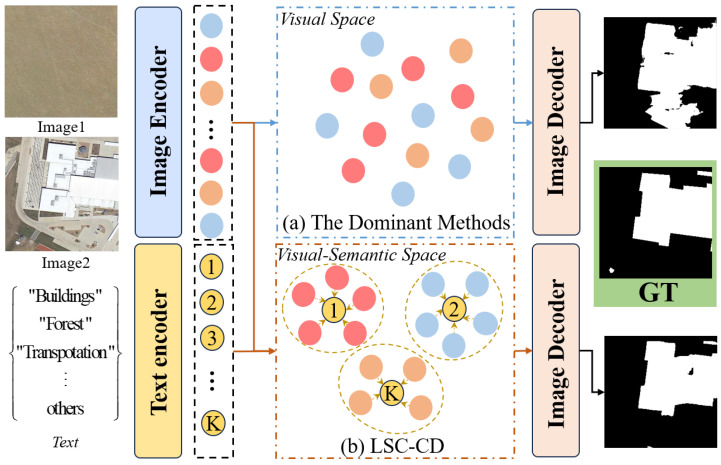
Structural comparison between semantic-agnostic dominant methods [26,28,29,34,36,37] and the proposed LSC-CD. Compared to the disorder of the dominant methods in visual space, the LSC-CD obtains more orderly and compact semantic embeddings in visual-semantic space through clustering.

Despite the demonstrated success of these methods, researchers have only used a semantic-agnostic binary ground-truth mask as supervision to train their model, which may suffer from land cover classification ambiguity issues. This is due to the fact that in highly complicated and dynamic varying environment scenarios, remote sensing images often contain hardly classified regions consisting of a variety of different land cover types with similar color or texture appearances, especially for regions across land cover boundaries. As shown in Figure 1, the input images contain interferences of changed land cover in different categories but have similar texture and color appearances. This is prone to causing the visual representations of the different land cover types learned by these methods to be indistinguishable in the visual space, leading to irrelevant pseudo-changes in the change mask (see Figure 1a). To address this issue, it is necessary to explore an effective framework for learning more discriminative representations in RSCD.

Recently, vision-language representation learning has gained significant attention in computer vision [38,39,40], focusing on learning robust representations from image–text pairs. This paradigm introduces multimodal information to assist visual representation learning. Following this paradigm, the widely adopted CLIP [38] uses a contrastive learning strategy on large-scale image–text pairs to project the text and visual features into the same latent space, termed as visual-semantic space. The CLIP has demonstrated outstanding zero-shot generalization for various downstream tasks, such as Text4point [41], Gridclip [42], and Clip2scene [43]. This motivated us to utilize the pretrained CLIP to construct visual-semantic space to assist in representation learning in RSCD.

To this end, this paper presents a language-guided semantic clustering framework for RSCD, which uses clustering with semantic embeddings to modulate change region embeddings, effectively categorizing different types of changes in visual-semantic space. Specifically, the text superior memory bank is first constructed based on common classes in RSCD datasets, and the CLIP is used to map this memory bank into semantic embeddings. Meanwhile, bi-temporal images are encoded into changed region embeddings through an encoder [44]. Then, a lightweight CLIP adapter module (CAM) is designed to modulate the semantic embeddings, aligning them adaptively with the changed region embeddings. Next, the semantic clustering module (SCM) is applied to group the change region embeddings around the semantic embeddings. Finally, a lightweight decoder decodes the modulated change region embeddings, with the entire process constrained by a loss function. Extensive experiments on three public benchmarks including LEVIR-CD [45], WHU-CD [23], and SYSU-CD [46] demonstrate the superiority of the proposed LSC-CD over various state-of-the-art methods.

The main contributions of this paper can be summarized as follows:

- We propose a language-guided semantic clustering framework for RSCD that achieves strong semantic modeling capabilities by introducing the CLIP.
- We design an SCM that modulates change embeddings from a semantic-guided perspective using semantic clustering.
- We develop a CAM to fine-tune the CLIP text encoder, aligning its encoded semantic embeddings to the change region embeddings.

## 2. Related Work

This section primarily explores the recent research related to our proposed method, focusing on two key components: semantic segmentation and RSCD. In semantic segmentation, we primarily discuss relevant studies in the field of computer vision, which inspired our approach. In RSCD, we review the existing mainstream change detection methods, highlighting their strengths and limitations, and introduce the research gap to emphasize the significance of our proposed method.

### 2.1. Semantic Segmentation

Semantic segmentation is a fundamental task in computer vision, aiming to assign a class label to every pixel in an image. RSCD is an important downstream task of semantic segmentation. Currently, most semantic segmentation models follow the structure of FCN [47], which employs an encoder–decoder framework. Subsequent works have introduced various optimizations. For example, some studies [48,49,50] have replaced the encoder with architectures from classification tasks, such as VGG [51], ResNet [52], and HRNet [53]. With the emergence of transformer [35], several works [54,55,56,57] have incorporated attention mechanisms to enhance the ability to capture long-range dependencies. Broadly, these methods can be classified into two types: the class-label-based semantic segmentation and the pretrained semantic segmentation models.

The class-label-based semantic segmentation models [58,59,60,61,62] rely on large amounts of pixel-level annotations as supervision, using encoder–decoder structures to directly learn semantic information from images in a data-driven manner. However, collecting pixel-level annotations requires significant resources. To address this issue, pretrained semantic segmentation methods have emerged. These methods [63,64,65,66,67,68] typically leverage pretrained weights to assist semantic segmentation, significantly reducing the need for extensive pixel-level annotations and cutting costs. However, the performance of these methods is often limited by the pretrained weights. The early approaches [63,64,65] mostly utilized the large-scale ImageNet [69] classification datasets as pretrained data, using image-level labels to aid semantic presentation learning. Due to the limitations of ImageNet data, models pr-trained on ImageNet often exhibit incomplete semantic modeling capabilities for segmentation tasks. The emergence of large-scale models such as the CLIP [38] has partially mitigated these challenges. Subsequent works [66,67,68] have begun applying large-scale models to semantic segmentation tasks, achieving superior performance.

In this work, we applied the CLIP to RSCD. Specifically, we leveraged the pretrained CLIP text encoder to encode semantic information and used a lightweight and effective CLIP adapter module to align the semantic information with the changed features.

### 2.2. Remote Sensing Change Detection

Remote sensing change detection methods can be categorized into traditional methods and deep learning-based methods.

Traditional methods include algebra-based methods, statistics-based methods, and trans -formation-based methods [70]. Algebra-based methods generate a change map using techniques such as image difference [10], image regression [11], image ratioing [12], and change vector analysis (CVA) [13]. The performance of these methods relies heavily on the selection of empirical thresholds. Statistics-based methods analyze the statistical properties of the image or parts of it, deriving a change map based on pixel distribution and the corresponding attributes. However, the effectiveness of these methods is constrained by the reliability of the statistical properties. Transformation-based methods employ techniques such principal component analysis (PCA) [14], multivariate alteration detection (MAD) [15], Gramm–Schmidt transformation [16], and tasseled cap transformation [17] to transform the images in a way that enhances change regions while suppressing unchanged areas. Nonetheless, these methods still depend on empirical thresholds. For example, PCA requires manually set parameters, limiting its ability to adaptively learn changed features. Overall, these traditional methods are overly reliant on handcrafted features, lack robustness, and struggle to effectively address the increasingly complex scenarios in remote sensing change detection.

Existing state-of-the-art methods are mostly based on deep learning in RSCD, which breaks through the limitations of traditional methods by adopting a data-driven strategy to adaptively learn changed features. Throughout the development of deep learning technologies, two main paradigms have emerged: CNN-based methods and transformer-based methods. The CNN-based methods leverage the powerful nonlinear modeling capabilities of convolutional neural networks (CNNs) to directly extract changed features from bi-temporal images and decode a corresponding change map, breaking the limitations of traditional handcrafted feature-based methods. Wang et al. [19] introduced faster region-based convolutional neural network (Faster R-CNN) into change detection and achieved notable success. Amin et al. [20] further improved performance by incorporating a Siamese architecture into CNN-based approaches. Building on this, Daudt et al. [18] proposed three fully convolutional change detection models that refined the Siamese structure. Liu et al. [21] combined the UNet [71] architecture with Siamese networks, interpreting change detection as an image translation problem. Fang et al. [25] optimized the fusion of deep and shallow features in the Siamese framework, designing the ECAM module to guide the model in learning effective representations. Yin et al. [22] introduced attention mechanisms into CNN-based models, further enhancing their performance. The aforementioned CNN-based methods have significantly advanced remote sensing change detection, but they are limited by their lack of long-range modeling capabilities, particularly in complex scenarios.

To overcome this limitation, transformer-based methods, recognized for their superior long-range modeling abilities, have emerged as promising solutions in bi-temporal remote sensing change detection problems. Chen et al. [28] introduced a transformer encoder to model spatial contexts based on semantic tokens, enhancing long-range modeling for bi-temporal remote sensing change detection. Yu et al. [34] proposed a global context-aware transformer structure, improving global context learning to address bi-temporal remote sensing change detection. Bandara et al. [36] designed a transformer-based Siamese network architecture, incorporating multi-scale change features to solve the bi-temporal remote sensing change detection problem. Zhang et al. [33] combined a transformer-based Siamese network with a UNet architecture to extract effective global information for this problem. Li et al. [30] proposed a hybrid transformer structure, aiming to leverage the strengths of both CNNs and transformers to learn local-global context features for bi-temporal remote sensing change detection.

While transformer-based methods have broken through the inherent limitations of CNNs due to their superior long-range modeling capabilities, they often rely on binary masks as supervision, lacking effective semantic label guidance. As a result, the semantic representations learned by these methods are constrained. To address this, we propose a semantic-guided transformer-based Siamese network. By introducing semantic clustering centers at the input stage, our approach guides semantic representation learning, compensating for the absence of semantic label guidance at the output stage.

## 3. Methods

This section presents the technical details of the proposed LSC-CD, which consists of four main sections: Overview, CLIP-Adapter Module, Semantic Clustering Module, and Loss Function. In the Overview Section, we introduce the overall pipeline of LSC-CD. In the CLIP-Adapter Module and Semantic Clustering Module Section, we begin by explaining the motivation behind the design of each module, followed by a detailed description of their implementation. In the Loss Function Secction, we explain the supervised loss function we developed for LSC-CD, based on supervised RSCD.

### 3.1. Overview

**Figure 2:**
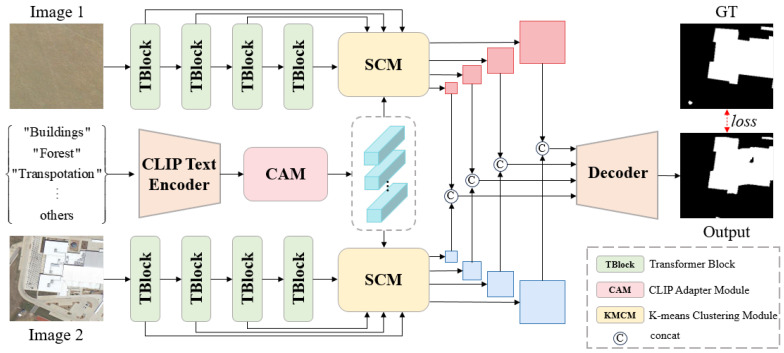
The pipeline of the proposed LSC-CD. The Transformer Block is the multi-scale transformer encoder from Segformer [44].

Figure 2 illustrates the pipeline of the proposed LSC-CD. First, the bi-temporal images $\left\{I_{1},I_{2}\right\}\in R^{H\times W\times 3}$ are encoded into multi-scale change features ${\left\{F_{1}^{i},F_{2}^{i}\right\}}_{i=1}^{4}$ using a Segformer encoder [44], where *H* denotes the height of the image, *W* denotes the width of the image, and *i* refers to the *i*-th scale of the features. Meanwhile, the text superior memory bank $T=\left\{t_{1},t_{2},\cdots ,t_{k}\right\}$ is encoded into semantic embeddings $S\in R^{K\times C}$ through a pretrained CLIP text encoder, where *K* denotes the number of prior texts, and *C* represents the channel dimension. Next, the CAM is applied to adaptively modulate the semantic embeddings S to generate the modulated embeddings $\hat{S}\in R^{K\times C}$. Then, $\hat{S}$ and $\left\{F_{1}^{i},F_{2}^{i}\right\}$ are fed into the SCM, where $\hat{S}$ serve as clustering centers to modulate $\left\{F_{1}^{i},F_{2}^{i}\right\}$, resulting in the modulated change embeddings $\left\{{\hat{F}}_{1}^{i},{\hat{F}}_{2}^{i}\right\}$. Finally, the concatenated $\left\{{\hat{F}}_{1}^{i},{\hat{F}}_{2}^{i}\right\}$ is passed through a lightweight decoder [36] to generate a change map.

### 3.2. CLIP-Adapter Module (CAM)

To obtain semantic information that guides the change detection model, we designed a CAM based on CLIP, aiming to directly incorporate the outstanding semantic knowledge from the CLIP into RSCD. In the CAM, considering the complexity of the RSCD scenario, we encounter two challenges. First, it is impractical to anticipate all possible text descriptions of subcategories. Second, overly detailed subcategories may be subject to the fluctuations influenced by environmental factors. For example, the distinction between dense forests and sparse forests may arise from seasonal changes rather than real changes that have occurred. Consequently, fine-grained prior change detection text memory may not necessarily benefit the change detection model in identifying changes. Therefore, we establish a coarse-grained text memory bank T, which is described in Table 1, and use a pretrained CLIP text encoder to encode it into corresponding semantic embeddings. In the encoding process, we focus solely on encoding the category information while avoiding the detailed encoding of each subcategory to prevent excessive description. This process can be formalized as

(1) $$S_{k}=Encoder_{t}\left(t_{k}\right),$$

where $Encoder_{t}$ represents the text encoder of the CLIP, $S_{k}\in R^{1\times C}$ denotes the semantic embedding corresponding to the *k*-th text, and *k* represents the index of the coarse-grained text memory bank T.

Although the CLIP can directly encode semantic information by introducing a coarse-grained text memory bank T, there is a misalignment between the semantic embeddings S and the change embeddings $\left\{F_{1}^{i},F_{2}^{i}\right\}$. To align the semantic embeddings from the CLIP with the change embeddings, we designed a lightweight CLIP adapter module that adaptively modulates the encoded semantic embeddings to align with the change embeddings. The CLIP adapter module consists of two layers of MLP [72] and a residual structure [52]. This process can be formulated as

(2) $$\hat{S}=\alpha Relu\left(SW_{1}\right)W_{2}+\left(1-\alpha \right)S,$$

where Relu(·) denotes the ReLU function [73], $\hat{S}\in R^{K\times C}$ represents the modulated semantic embeddings, *K* denotes the length of the text memory, *C* denotes channel dimension. $W_{1}$, $W_{2}$ denote the adaptive weights of the MLP [72], and α signifies the residual ratio, which is set to 0.7.

### 3.3. Semantic Clustering Module (SCM)

**Figure 3:**
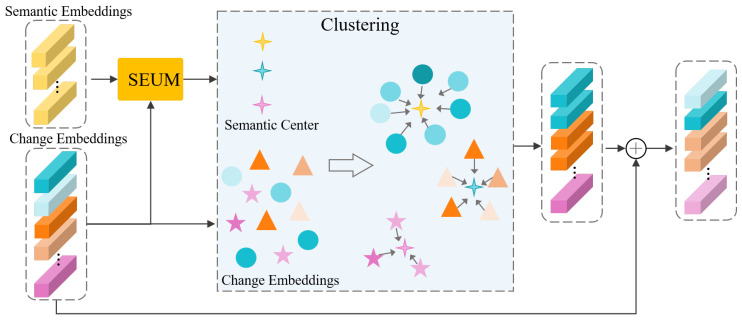
Architecture of the proposed SCM.

**Figure 4:**
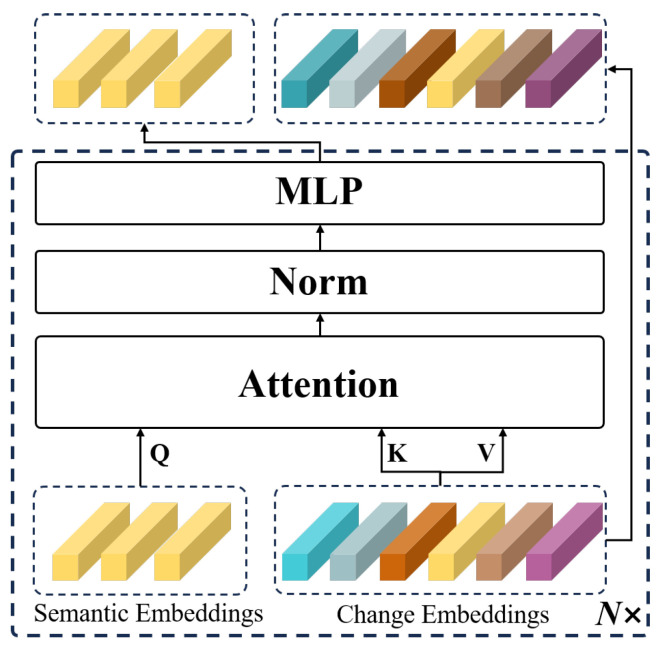
Architecture of the SEUM in the SCM.

To fully integrate the modulated semantic embeddings $\hat{S}$ obtained from the CAM with the change embeddings $\left\{F_{1}^{i},F_{2}^{i}\right\}$, we designed the SCM based on clustering, as illustrated in Figure 3. In SCM, the semantic embeddings $\hat{S}$ serve as clustering centers, while the $\left\{F_{1}^{i},F_{2}^{i}\right\}$ are treated as the features to be clustered. Given that we utilize a coarse-grained text memory bank T, there may be misalignment between the semantic embeddings of categories and the visual embeddings of specific subcategories. To address this issue, we developed the SEUM to adjust the $\hat{S}$ within the SCM. The structure of the SEUM is illustrated in Figure 4.

In the SEUM, we designed an interactive attention mechanism that utilizes the change embeddings $\left\{F_{1}^{i},F_{2}^{i}\right\}$ to update the $\hat{S}$. This process can be formalized as

(3) $${\hat{S}}_{j}^{i}=MLP\left(Norm\left(Attention\left(\hat{S},F_{j}^{i},F_{j}^{i}\right)\right)\right),$$

(4) $$Attention\left(Q,K,V\right)=softmax\left(QK^{T}\right)V,$$

(5) $$softmax\left(z_{i}\right)=\frac{exp(z_{i})}{\sum_{j}exp\left(z_{j}\right)},$$

where Attention(·) represents the cross-attention mechanism, and Norm(·) denotes normalization [35]. MLP(·) refers to a single-layer multi-layer perceptron [72]. softmax(·) represents the activation function, $i\in \left\{1,2,3,4\right\}$, and $j\in \left\{1,2\right\}$.

After the SEUM, to fuse the semantic information and change embeddings $\left\{F_{1}^{i},F_{2}^{i}\right\}$, we use cosine similarity as the metric to cluster $\left\{F_{1}^{i},F_{2}^{i}\right\}$, with the modulated semantic embeddings $\hat{S}$ serving as the clustering centers. This process can be expressed as

(6) $$F_{j}^{i}=\beta sim\left(F_{j}^{i},{\hat{S}}_{j}^{i}\right)*{S_{j}^{i}}^{T}+\left(1-\beta \right)F_{j}^{i},$$

(7) $$sim\left(A,B\right)=\frac{AB}{\left|A\right|\left|B\right|},$$

where β is the modulation ratio, set to 0.5. sim(·,·) represents the cosine similarity.

### 3.4. Loss Funcation

The proposed LSC-CD is a supervised change detection method, where the entire learning process is guided by binary semantic-agnostic masks. During training, we employ the binary cross-entropy loss $\ell_{bce}$ [74] to supervise the learning of changed features.

In this paper, the binary cross-entropy loss can be formalized as

(8) $$\ell_{BCE}=\frac{1}{H\times W}\sum_{i}^{H}\sum_{j}^{W}\left[y_{i}\log{\hat{y}}_{i}+\left(1-y_{i}\right)\log\left(1-{\hat{y}}_{i}\right)\right],$$

where *H* and *W* represent the height and width of the image, respectively; $y_{i}$ denotes the ground truth (GT) label of the *i*-th pixel, and ${\hat{y}}_{i}$ refers to the predicted value for the *i*-th pixel in the LSC-CD output.

## 4. Experiments

This section primarily describes the experimental process, including six subsections: Datasets, Evaluation Metrics, Implementation Details, Quantitative Analysis, Qualitative Analysis, and Ablation Studies. In the Datasets Section, we provide a comprehensive description of three public datasets. In the Evaluation Metrics Section, we present five metrics used to assess the experimental results. In the Implementation Details Section, we outline the experimental environment and the settings for hyperparameters. In the Quantitative Analysis Section, we analyze the differences between our method and other methods from a qualitative perspective. In the Qualitative Analysis Section, we quantitatively examine the differences between our method and other methods, incorporating the evaluation metrics. Finally, in the Ablation Studies Section, we analyze the impact of different modules on our method.

### 4.1. Datasets

**LEVIR-CD.** The LEVIR-CD is a large-scale remote sensing dataset for building change detection models, released by Beihang University in 2020. It consists of 637 pairs of very-high-resolution Google Earth images with a resolution of 1024 × 1024 pixels, where each pixel represents 0.2 m. The dataset primarily focuses on building changes, including building growth and demolition, with change areas labeled as 1 and unchanged areas labeled as 0, without semantic labels. It contains many irrelevant changes due to seasonal variations. The buildings in LEVIR-CD range from villas, small garages, and high-rise apartments to large warehouses, with a total of 31,333 changed buildings. Following [36], we split the original data into 256 × 256 patches without overlap, resulting in 10,192 images, of which 7120 are used for training, 2048 for testing, and 1024 for validation.
**WHU-CD.** The WHU-CD is another building change detection dataset for remote sensing, similar to LEVIR-CD. It contains two aerial images with a resolution of 32,507 × 15,354 pixels, where each pixel represents 0.3 m. Like LEVIR-CD, it does not provide additional semantic labels. This dataset has been widely used as a benchmark for change detection studies. However, due to the large resolution of the image pairs, they need to be divided into smaller patches for model input, and there is no standard method for this splitting. To ensure a fair comparison, we followed [37] for splitting the dataset. Specifically, we divided the original images into 256 × 256 pixel patches, resulting in 7620 image pairs. Of these, 6096 pairs are used for training, 762 pairs for testing, and 762 pairs for validation.
**SYSU-CD.** The SYSU-CD contains 20,000 pairs of high-resolution images, each with 256 × 256 pixels, collected in Hong Kong between 2007 and 2014, where each pixel represents 0.5 m. Unlike LEVIR-CD and WHU-CD, SYSU-CD includes a variety of changes, including newly built urban buildings, suburban expansion, groundwork before construction, vegetation changes, road expansion, and sea construction. Change areas are labeled as 1 and unchanged areas as 0, without semantic labels. The dataset consists of 12,000 pairs for training, 4000 pairs for testing, and 4000 pairs for validation.

### 4.2. Evaluation Metrics

In the experiment, we adopted five common evaluation metrics to measure the model’s ability to segment changed regions, including precision (Pr), recall (Re), overall accuracy (OA), F1 score (F1), and the intersection over union (IoU). Among these, F1 is used as the primary evaluation metric, with the other four metrics being considered in conjunction with F1. These five metrics can be formulated as follows:

(9) $$Pr=\frac{TP}{TP+FP},$$

(10) $$Re=\frac{TP}{TP+FN},$$

(11) $$OA=\frac{TP+TN}{TP+FP+TN+FN},$$

(12) $$F1=\frac{2\times Pr\times Re}{Pr+Re},$$

(13) $$IoU=\frac{TP}{TP+FP+FN},$$

where TP represents true positives, indicating the number of pixels correctly predicted as changed. FP represents false positives, indicating the number of pixels incorrectly predicted as changed when no change occurred. TN represents true negatives, indicating the number of pixels correctly predicted as unchanged. FN represents false negatives, indicating the number of pixels incorrectly predicted as unchanged when a change occurred.

### 4.3. Implementation Details

We deployed our proposed LSC-CD within a PyTorch framework and used an NVIDIA RTX 3090 GPU. The RTX 3090 is designed and manufactured by NVIDIA Corporation, whose headquarters are located in Santa Clara, CA, USA. During the training, to ensure fairness, we applied four commonly used data augmentation techniques: random flips, Gaussian blurs, random cropping, and random color dithering. The network took 256 × 256 images as the input, with a batch size of 8. The image encoder utilized the Segformer encoder’s pretrained weights, while the rest of the network was randomly initialized. The entire process was supervised by a binary cross-entropy loss function. Adam [75] was used as the optimizer, with a learning rate of 0.0001, decaying by 0.00002 every 50 epochs, for a total of 200 training epochs.

### 4.4. Quantitative Comparison

To ensure fairness, we conducted comparisons on three public datasets: LEVIR-CD, WHU-CD, and SYSU-CD. We evaluated various methods across these datasets. Due to limitations regarding the availability of open-source code, for some methods, we directly referenced the results reported in the original papers. As a result, some data may be missing, and we marked these with the symbol “-”. We compared 15 different methods, including FC-EF [18], FC-Siam-Di [18], FC-Siam-Conc [18], STANet [23], IFNet [24], SNUNet-CD [25], BIT [28], TransUNetCD [30], ChangeFormer [36], StransUNet [31], ICIF-Net [29], AMCA [32], DMINet [26], GCD-DDPM [37], and GCFormer [34], with GCD-DDPM and GCFormer being the latest methods.

Table 2 lists the performance of the various change detection methods on the LEVIR-CD dataset. The results showed that our proposed LSC-CD achieved state-of-the-art performance. LSC-CD attained an F1 score of 92.01, which was the best performance. In addition, LSC-CD performed well in terms of precision, IoU, and overall accuracy. It is worth noting that LSC-CD did not achieve the best performance in recall. This is because the recall metric considers both true positives and false negatives, and some change detection models tended to predict more background, which reduces false negatives and increased recall. However, This tendency does not align with practical demands, making recall alone an insufficient reflection of a change detection model’s true performance. Therefore, a comprehensive evaluation of all metrics is necessary.

Table 3 lists the performance of various change detection methods on the WHU-CD dataset. The results show that our proposed LSC-CD achieved the best performance in F1, recall, IoU, and overall accuracy, with an F1 score of 94.75. This demonstrates that the introduction of effective semantic modeling in LSC-CD led to outstanding results. Notably, LSC-CD only achieved the second-best performance in precision. Similar to recall, precision is influenced by true positives and false positives. A high precision indicates that the change detection model tends to predict more foreground, which can result in many irrelevant changes being detected. This tendency is evident in earlier methods like IFNet, which achieved the best precision but did not perform as well overall compared to current state-of-the-art methods. In contrast, LSC-CD delivered the best overall performance, further validating the effectiveness of our proposed approach.

Table 4 lists the comparative results of several methods on SYSU-CD. Since SYSU-CD is a multi-class change detection dataset, it is more challenging compared to LEVIR-CD and WHU-CD, as reflected in the performance metrics. Due to the unavailability of the code for some recent works, we only compared algorithms with accessible code. From the results of the comparison, similar to LEVIR-CD and WHU-CD, our proposed LSC-CD demonstrates outstanding performance across several metrics, achieving an F1 score of 81.29, achieves state-of-the-art performance. The strong performance on SYSU-CD indicates that the integration of CLIP semantic knowledge into LSC-CD significantly enhances the overall performance of the change detection model.

### 4.5. Qualitative Comparison

To ensure fairness, we conducted the qualitative comparison on three public datasets: LEVIR-CD, WHU-CD, and SYSU-CD, similar to the quantitative comparison. We visualized selected test samples from five different methods, including FC-EF [18], BIT [28], ChangeFormer [36], ICIFNet [29], and DMINet [26].

**Figure 5:**
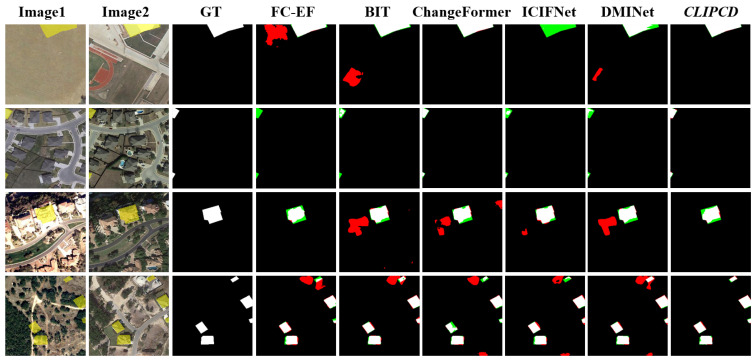
Qualitative comparison results of different CD methods on LEVIR-CD datasets: the black represents true negative, the white represents true positive, the red represents false positive and the green represents false negative.

Figure 5 demonstrates the qualitative results of the different methods on the LEVIR-CD dataset. LEVIR-CD is a building change detection dataset, where only building changes are considered changes, which can be observed from the ground truth binary mask (GT). This dataset primarily tests the robustness of change detection models in handling no-task-specific changes. It is apparent from the findings that our proposed LSC-CD yielded masks that were nearly identical to the ground truth binary masks (GT). Notably, LSC-CD showed the fewest false positives, as reflected by the smallest red areas in Figure 5. This demonstrates that by incorporating effective semantic guidance, LSC-CD can effectively suppress the interference of no-task-specific changes.

**Figure 6:**
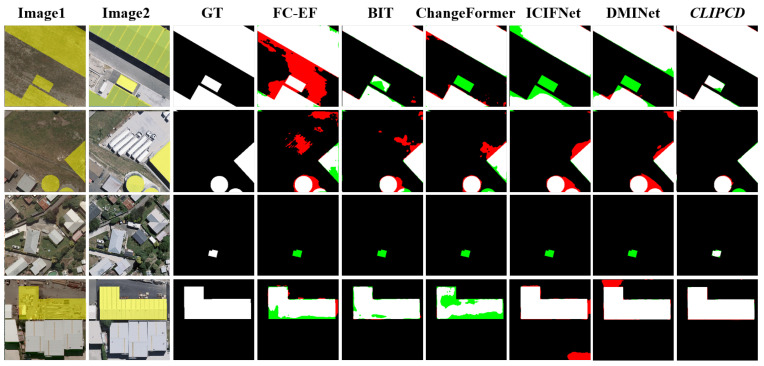
Qualitative comparison results of different CD methods on WHU-CD datasets: the black represents true negative, the white represents true positive, the red represents false positive and the green represents false negative.

Figure 6 illustrates a quantitative comparison of different methods on the WHU-CD dataset. The results show that our proposed LSC-CD predicted the fewest false positives while closely aligning with the ground truth, indicating that LSC-CD effectively suppresses no-task-specific changes.

**Figure 7:**
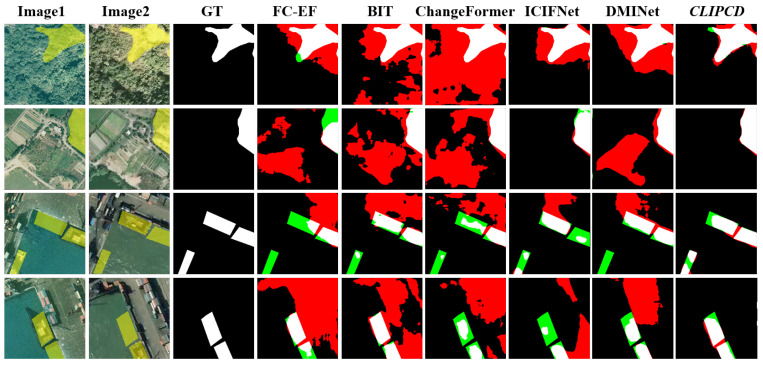
Qualitative comparison results of different CD methods on SYSU-CD datasets: the black represents true negative, the white represents true positive, the red represents false positive, and the green represents false negative.

Figure 7 illustrates a quantitative comparison of different methods on the SYSU-CD dataset. Unlike LEVIR-CD and WHU-CD, which focus on single-class change detection, SYSU-CD is a multi-class change dataset containing more irrelevant disturbances. The results show that other methods introduced a significant amount of no-task-specific changes. In contrast, our method effectively overcame this issue, demonstrating that LSC-CD, by incorporating semantic information to guide change feature learning, can effectively suppress no-task-specific changes in multi-class change scenarios.

### 4.6. Ablation Studies

To validate the effectiveness of the proposed CAM and SCM, we conducted ablation experiments on the LEVIR-CD dataset. We compared five variants: the baseline (ChangeFormer [36]), which used the same encoder and decoder as our approach; the baseline with the CLIP method; the baseline with the CLIP and CAM method; the baseline with the CLIP method, CAM, and SCM without the SEUM method; and the full LSC-CD method. The comparison results are listed in Table 5. From the results, the baseline with the CLIP method achieved an improvement in F1 and IoU scores by 0.44 and 0.74, respectively, compared to the baseline, demonstrating the importance of semantic information for the RSCD task. The baseline with the CLIP and CAM method further improved the F1 and IoU scores by 0.24 and 0.4, respectively, indicating that modulating semantic embeddings to align change embeddings is necessary. The method with SCM integrated the modulated semantic embeddings with the change embeddings, resulting in significant increases of 0.61 and 1.04 in F1 and IoU, respectively, showing that using coarse-grained clustering facilitates the fusion of semantic and change embeddings. Finally, the full LSC-CD, which incorporated the SEUM module to refine clustering centers, boosted the F1 and IoU scores by 0.32 and 0.49, respectively, indicating that the introduction of the SEUM module helps improve the RSCD performance.

**Figure 8:**
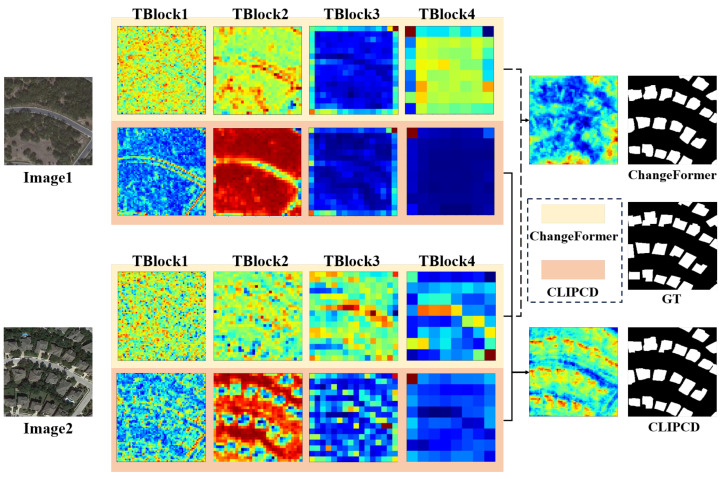
Heatmap comparison results. TBlock1–TBlock4 represent attention maps at four different scales from the encoder [44]. The 1st and 3rd rows show the pre-change and post-change heatmaps of the baseline (ChangeFormer), while the 2nd and 4th rows show the pre-change and post-change heatmaps of LSC-CD.

Figure 8 presents a comparison of the attention distributions between our proposed LSC-CD and ChangeFormer. The results indicate that our proposed LSC-CD, by introducing CAM and SCM to guide change feature clustering with semantic information, achieved a more refined attention distribution for both the heatmaps during the encoding process and the final concatenated heatmap compared to the baseline (ChangeFormer).

To the best of our knowledge, we are not the first to apply the CLIP to the RSCD task. Before the development of LSC-CD, ChangeCLIP [76] was another viable approach. Both methods share the use of the prior text memory bank but differ in that, on the one hand, the proposed LSC-CD uses only coarse-grained categories as clustering centers without the need for fine-grained text prompts. On the other hand, LSC-CD incorporates semantic guidance for change embeddings encoding through clustering in the encoder without fusing at the decoder. Table 6 compares the performance of both methods, using the same CLIP, on the LEVIR-CD dataset. The results demonstrate that LSC-CD generally outperforms ChangeCLIP, validating the effectiveness of introducing semantic information into the encoder.

## 5. Conclusions

In this paper, we proposed the LSC-CD, a language-guided semantic cluster framework for RSCD. First, we introduced a category text memory bank based on dataset statistics. Then, the CLIP text encoder was utilized to transform the bank into the corresponding semantic embeddings. Afterward, we designed the CAM to fine-tune the semantic embeddings to align with the change region embeddings encoded from the bi-temporal images. Next, we developed the SCM to cluster the change region embeddings around the semantic embeddings in the visual-semantic space. Finally, we introduced a lightweight decoder to decode the change region embeddings after the SCM to yield the change mask prediction. The promising performance on three public RSCD benchmarks demonstrated that language-guided semantic clustering helps RSCD to distinguish noisy masks.

Although the proposed LSC-CD achieved excellent performance to a certain extent, we used additional text semantic information and introduced extra parameters. This kind of text semantic information needs to be provided in advance. We plan to utilize weak semantic segmentation to extract reliable semantic information from the bi-temporal images in a lightweight manner without additional information.

## Figures and Tables

**Table 1:** Common categories and subcategories of remote sensing images.

Category	Subcategory
Buildings	Residential buildings, Commercial buildings, Factory buildings,
	Church, Building
Forest	Tree, Forest Regrowth, Riparian Forest, Planted Forest,
	Evergreen Trees, Deciduous Trees, Sparse Forest, Dense Forest
Transportation	Truck, Cars, Ship, Trains, Airplane
Surface water	Lake, Sea, River, Reservoir, Wetlands, Canal
Roadway	Bridge, Freeway, Harbor, Runway, Railway, Road, Highway.
Sports	Basketball Court, Ground Track Field, Stadium, Tennis Court, Golf Course
Others	Container, Island, Snow Land, Pond, Fertile Land, Beach,
	Square, Parking Lot, Park

**Table 2:** The quantitative comparison of results of different methods on the LEVIR-CD dataset.

Method	Year	Structure	P ↑	R ↑	F1 ↑	IoU ↑	OA ↑
FC-EF [18]	2018	CNN	86.37	83.54	82.35	71.80	96.97
FC-Siam-Di [18]	2018	CNN	89.53	83.31	86.31	75.92	98.67
FC-Siam-Conc [18]	2018	CNN	91.99	76.77	83.69	71.96	98.49
STANet [23]	2020	CNN+Attention	83.81	91.00	87.26	77.40	98.66
IFNet [24]	2020	CNN	83.77	80.32	82.29	70.97	98.61
SNUNet-CD [25]	2021	CNN + Attention	89.18	87.17	88.16	78.83	98.82
BIT [28]	2021	CNN + Transformer	89.24	89.37	89.31	80.68	98.62
TransUNetCD [30]	2022	CNN + Transformer	92.43	89.82	91.11	83.67	-
ChangeFormer [36]	2022	Transformer	92.05	88.80	90.40	82.48	99.04
StransUNet [31]	2022	CNN + Transformer	92.30	90.55	91.41	84.19	99.13
ICIF-Net [29]	2023	CNN + Transformer	87.79	80.88	83.65	71.89	98.73
AMCA [32]	2023	CNN + Transformer	91.82	90.67	91.48	83.39	98.73
DMINet [26]	2023	CNN	92.52	89.94	90.71	82.99	99.07
GCD-DDPM [37]	2024	CNN	90.68	91.24	90.96	83.56	99.14
GCFormer [34]	2024	CNN + Transformer	89.34	92.50	90.83	83.21	99.08
LSC-CD	-	Transformer	93.31	90.69	92.01	85.15	99.19

Red represents the best performance, blue represents the second-best performance, and all metrics are expressed as percentages. The arrow ↑ indicates that a larger value corresponds to better performance.

**Table 3:** A quantitative comparison of the results of different methods on the WHU-CD dataset.

Method	Year	Structure	P ↑	R ↑	F1 ↑	IoU ↑	OA ↑
FC-EF [18]	2018	CNN	83.50	86.33	84.89	73.74	98.87
FC-Siam-Di [18]	2018	CNN	90.86	84.69	87.67	78.04	99.13
FC-Siam-Conc [18]	2018	CNN	84.02	87.72	85.83	75.18	98.94
STANet [23]	2020	CNN+Attention	79.37	85.50	82.32	69.95	98.52
IFNet [24]	2020	CNN	96.91	73.19	83.40	71.52	98.83
SNUNet-CD [25]	2021	CNN + Attention	85.60	81.49	83.50	71.67	98.71
BIT [28]	2021	CNN + Transformer	82.04	89.74	85.71	75.00	98.62
TransUNetCD [30]	2022	CNN + Transformer	93.59	89.60	91.60	84.42	-
ChangeFormer [36]	2022	Transformer	91.83	88.02	89.88	81.63	99.12
StransUNet [31]	2022	CNN + Transformer	93.21	90.15	91.65	84.59	99.29
ICIF-Net [29]	2023	CNN + Transformer	90.79	87.58	89.16	80.43	99.01
AMCA [32]	2023	CNN + Transformer	91.37	81.90	86.38	75.13	95.97
DMINet [26]	2023	CNN	93.84	86.25	88.69	79.68	98.97
GCD-DDPM [37]	2024	CNN	92.79	92.26	92.54	86.52	99.39
GCFormer [34]	2024	CNN	91.81	90.15	90.97	83.43	99.29
LSC-CD	-	Transformer	96.32	93.18	94.75	89.98	99.59

Red represents the best performance, blue represents the second-best performance, and all metrics are expressed as percentages. The arrow ↑ indicates that a larger value corresponds to better performance.

**Table 4:** The quantitative comparison of the results of different methods on the SYSU-CD dataset.

Method	Year	Structure	P ↑	R ↑	F1 ↑	IoU ↑	OA ↑
FC-EF [18]	2018	CNN	76.47	75.17	75.81	61.04	88.69
FC-Siam-Di [18]	2018	CNN	76.28	75.30	75.79	61.01	88.65
FC-Siam-Conc [18]	2018	CNN	73.67	76.75	75.18	60.23	88.05
IFNet [24]	2020	CNN	82.44	72.38	76.38	61.85	-
SNUNet-CD [25]	2021	CNN + Attention	83.58	75.87	79.54	66.02	90.79
BIT [28]	2021	CNN + Transformer	81.42	77.90	78.32	64.37	90.26
TransUNetCD [30]	2022	CNN + Transformer	82.59	77.73	80.09	66.79	90.88
ChangeFormer [36]	2022	Transformer	81.70	72.38	76.76	62.29	76.76
ICIF-Net [29]	2023	CNN + Transformer	83.37	78.51	80.74	68.12	91.24
LSC-CD	-	Transformer	88.64	75.06	81.29	68.47	91.85

Red represents the best performance, blue represents the second-best performance, and all metrics are expressed as percentages. The arrow ↑ indicates that a larger value corresponds to better performance.

**Table 5:** Results of ablation experiments on the LEVIR-CD dataset.

Method	P ↑	R ↑	F1 ↑	IoU ↑	OA ↑
baseline	92.05	88.80	90.40	82.48	99.04
baseline + CLIP	91.78	89.92	90.84	83.22	99.08
baseline + CLIP + CAM	91.36	90.80	91.08	83.62	99.09
baseline + CLIP + CAM + SCM	93.44	90.08	91.69	84.66	99.16
baseline + CLIP + CAM + SCM + SEUM	93.31	90.69	92.01	85.15	99.19

Red represents the best performance, blue represents the second-best performance, and all metrics are expressed as percentages. The arrow ↑ indicates that a larger value corresponds to better performance.

**Table 6:** Comparison between LSC-CD and ChangeCLIP.

Method	Year	P ↑	R ↑	F1 ↑	IoU ↑	OA ↑
ChangeCLIP(ViT-B/16)	2024	93.68	89.04	91.30	83.99	99.14
LSC-CD(ViT-B/16)	2024	93.31	90.69	92.01	85.15	99.19

Red represents the best performance, and all metrics are expressed as percentages. The arrow ↑ indicates that a larger value corresponds to better performance.

## Data Availability

The data presented in this study are available upon request from the corresponding author.

## References

[1] Qin D., Zhou X., Zhou W., Huang G., Ren Y., Horan B., He J., Kito N. (2018). MSIM: A change detection framework for damage assessment in natural disasters. Expert Syst. Appl..

[2] Michel U., Thunig H., Ehlers M., Reinartz P. (2012). Rapid change detection algorithm for disaster management. ISPRS Ann. Photogramm. Remote Sens. Spat. Inf. Sci..

[3] Zheng Z., Zhong Y., Wang J., Ma A., Zhang L. (2021). Building damage assessment for rapid disaster response with a deep object-based semantic change detection framework: From natural disasters to man-made disasters. Remote Sens. Environ..

[4] Bolorinos J., Ajami N.K., Rajagopal R. (2020). Consumption change detection for urban planning: Monitoring and segmenting water customers during drought. Water Resour. Res..

[5] Du P., Liu S., Gamba P., Tan K., Xia J. (2012). Fusion of difference images for change detection over urban areas. IEEE J. Sel. Top. Appl. Earth Obs. Remote Sens..

[6] Liu X., Lathrop R. (2002). Urban change detection based on an artificial neural network. Int. J. Remote Sens..

[7] Tarimo B., Mtalo E., Liwa E. (2013). Land use change detection and impact assessment on an agricultural area. J. Sustain. Dev..

[8] Prishchepov A.V., Radeloff V.C., Dubinin M., Alcantara C. (2012). The effect of Landsat ETM/ETM+ image acquisition dates on the detection of agricultural land abandonment in Eastern Europe. Remote Sens. Environ..

[9] Malinverni E.S., Rinaldi M., Ruggieri S. (2012). Agricultural crop change detection by means of hybrid classification and high resolution images. EARSeL EProc..

[10] Bruzzone L., Prieto D.F. (2000). Automatic analysis of the difference image for unsupervised change detection. IEEE Trans. Geosci. Remote Sens..

[11] Luppino L.T., Bianchi F.M., Moser G., Anfinsen S.N. (2019). Unsupervised image regression for heterogeneous change detection. arXiv.

[12] Ayhan B., Kwan C., Zhou J. (2018). A new nonlinear change detection approach based on band ratioing. Proceedings of the Algorithms and Technologies for Multispectral, Hyperspectral, and Ultraspectral Imagery XXIV.

[13] Chen J., Gong P., He C., Pu R., Shi P. (2003). Land-use/land-cover change detection using improved change-vector analysis. Photogramm. Eng. Remote Sens..

[14] Deng J., Wang K., Deng Y., Qi G. (2008). PCA-based land-use change detection and analysis using multitemporal and multisensor satellite data. Int. J. Remote Sens..

[15] Nielsen A.A. (2007). The regularized iteratively reweighted MAD method for change detection in multi-and hyperspectral data. IEEE Trans. Image Process..

[16] Hashim F., Dibs H., Jaber H.S. (2022). Adopting gram-schmidt and brovey methods for estimating land use and land cover using remote sensing and satellite images. Nat. Environ. Pollut. Technol..

[17] Han T., Wulder M.A., White J.C., Coops N.C., Alvarez M., Butson C. (2007). An efficient protocol to process Landsat images for change detection with tasselled cap transformation. IEEE Geosci. Remote Sens. Lett..

[18] Daudt R.C., Le Saux B., Boulch A. (2018). Fully convolutional siamese networks for change detection. Proceedings of the 2018 25th IEEE international conference on image processing (ICIP).

[19] Wang Q., Zhang X., Chen G., Dai F., Gong Y., Zhu K. (2018). Change detection based on Faster R-CNN for high-resolution remote sensing images. Remote Sens. Lett..

[20] El Amin A.M., Liu Q., Wang Y. (2016). Convolutional neural network features based change detection in satellite images. Proceedings of the First International Workshop on Pattern Recognition.

[21] Liu T., Li Y., Xu L. (2016). Dual-channel convolutional neural network for change detection of multitemporal SAR images. Proceedings of the 2016 International Conference on Orange Technologies (ICOT).

[22] Yin M., Chen Z., Zhang C. (2023). A CNN-Transformer Network Combining CBAM for Change Detection in High-Resolution Remote Sensing Images. Remote Sens..

[23] Chen H., Shi Z. (2020). A spatial-temporal attention-based method and a new dataset for remote sensing image change detection. Remote Sens..

[24] Zhang C., Yue P., Tapete D., Jiang L., Shangguan B., Huang L., Liu G. (2020). A deeply supervised image fusion network for change detection in high resolution bi-temporal remote sensing images. ISPRS J. Photogramm. Remote Sens..

[25] Fang S., Li K., Shao J., Li Z. (2021). SNUNet-CD: A densely connected Siamese network for change detection of VHR images. IEEE Geosci. Remote Sens. Lett..

[26] Feng Y., Jiang J., Xu H., Zheng J. (2023). Change detection on remote sensing images using dual-branch multilevel intertemporal network. IEEE Trans. Geosci. Remote Sens..

[27] LeCun Y., Boser B., Denker J.S., Henderson D., Howard R.E., Hubbard W., Jackel L.D. (1989). Backpropagation applied to handwritten zip code recognition. Neural Comput..

[28] Chen H., Qi Z., Shi Z. (2021). Remote sensing image change detection with transformers. IEEE Trans. Geosci. Remote Sens..

[29] Feng Y., Xu H., Jiang J., Liu H., Zheng J. (2022). ICIF-Net: Intra-scale cross-interaction and inter-scale feature fusion network for bitemporal remote sensing images change detection. IEEE Trans. Geosci. Remote Sens..

[30] Li Q., Zhong R., Du X., Du Y. (2022). TransUNetCD: A hybrid transformer network for change detection in optical remote-sensing images. IEEE Trans. Geosci. Remote Sens..

[31] Yuan J., Wang L., Cheng S. (2022). STransUNet: A siamese TransUNet-based remote sensing image change detection network. IEEE J. Sel. Top. Appl. Earth Obs. Remote Sens..

[32] Xu X., Yang Z., Li J. (2023). AMCA: Attention-guided multiscale context aggregation network for remote sensing image change detection. IEEE Trans. Geosci. Remote Sens..

[33] Zhang C., Wang L., Cheng S., Li Y. (2022). SwinSUNet: Pure transformer network for remote sensing image change detection. IEEE Trans. Geosci. Remote Sens..

[34] Yu W., Zhuo L., Li J. (2024). GCFormer: Global Context-aware Transformer for Remote Sensing Image Change Detection. IEEE Trans. Geosci. Remote Sens..

[35] Vaswani A. (2017). Attention Is All You Need. Advances in Neural Information Processing Systems.

[36] Bandara W.G.C., Patel V.M. (2022). A transformer-based siamese network for change detection. Proceedings of the IGARSS 2022–2022 IEEE International Geoscience and Remote Sensing Symposium.

[37] Wen Y., Ma X., Zhang X., Pun M.O. (2024). GCD-DDPM: A generative change detection model based on difference-feature guided DDPM. IEEE Trans. Geosci. Remote Sens..

[38] Radford A., Kim J.W., Hallacy C., Ramesh A., Goh G., Agarwal S., Sastry G., Askell A., Mishkin P., Clark J. Learning transferable visual models from natural language supervision. Proceedings of the International Conference on Machine Learning, PMLR.

[39] Gu T., Yang K., An X., Feng Z., Liu D., Cai W., Deng J. (2024). RWKV-CLIP: A Robust Vision-Language Representation Learner. arXiv.

[40] Lan M., Chen C., Ke Y., Wang X., Feng L., Zhang W. (2024). Clearclip: Decomposing clip representations for dense vision-language inference. arXiv.

[41] Huang R., Pan X., Zheng H., Jiang H., Xie Z., Wu C., Song S., Huang G. (2024). Joint representation learning for text and 3d point cloud. Pattern Recognit..

[42] Lin J., Gong S. (2023). Gridclip: One-stage object detection by grid-level clip representation learning. arXiv.

[43] Chen R., Liu Y., Kong L., Zhu X., Ma Y., Li Y., Hou Y., Qiao Y., Wang W. Clip2scene: Towards label-efficient 3d scene understanding by clip. Proceedings of the IEEE/CVF Conference on Computer Vision and Pattern Recognition.

[44] Xie E., Wang W., Yu Z., Anandkumar A., Alvarez J.M., Luo P. (2021). SegFormer: Simple and efficient design for semantic segmentation with transformers. Adv. Neural Inf. Process. Syst..

[45] Ji S., Wei S., Lu M. (2018). Fully convolutional networks for multisource building extraction from an open aerial and satellite imagery data set. IEEE Trans. Geosci. Remote Sens..

[46] Shi Q., Liu M., Li S., Liu X., Wang F., Zhang L. (2021). A deeply supervised attention metric-based network and an open aerial image dataset for remote sensing change detection. IEEE Trans. Geosci. Remote Sens..

[47] Long J., Shelhamer E., Darrell T. Fully convolutional networks for semantic segmentation. Proceedings of the IEEE Conference on Computer Vision and Pattern Recognition.

[48] Sugirtha T., Sridevi M. (2022). Semantic segmentation using modified u-net for autonomous driving. Proceedings of the 2022 IEEE International IOT, Electronics and Mechatronics Conference (IEMTRONICS).

[49] Seong S., Choi J. (2021). Semantic segmentation of urban buildings using a high-resolution network (HRNet) with channel and spatial attention gates. Remote Sens..

[50] Zhuang J., Yang J., Gu L., Dvornek N. Shelfnet for fast semantic segmentation. Proceedings of the IEEE/CVF International Conference on Computer Vision Workshops.

[51] Simonyan K., Zisserman A. (2014). Very deep convolutional networks for large-scale image recognition. arXiv.

[52] He K., Zhang X., Ren S., Sun J. Deep residual learning for image recognition. Proceedings of the IEEE Conference on Computer Vision and Pattern Recognition.

[53] Wang J., Sun K., Cheng T., Jiang B., Deng C., Zhao Y., Liu D., Mu Y., Tan M., Wang X. (2020). Deep high-resolution representation learning for visual recognition. IEEE Trans. Pattern Anal. Mach. Intell..

[54] Zheng S., Lu J., Zhao H., Zhu X., Luo Z., Wang Y., Fu Y., Feng J., Xiang T., Torr P.H. Rethinking semantic segmentation from a sequence-to-sequence perspective with transformers. Proceedings of the IEEE/CVF Conference on Computer Vision and Pattern Recognition.

[55] Strudel R., Garcia R., Laptev I., Schmid C. Segmenter: Transformer for semantic segmentation. Proceedings of the IEEE/CVF International Conference on Computer Vision.

[56] Chu X., Tian Z., Wang Y., Zhang B., Ren H., Wei X., Xia H., Shen C. (2021). Twins: Revisiting the design of spatial attention in vision transformers. Adv. Neural Inf. Process. Syst..

[57] Dosovitskiy A. (2020). An image is worth 16x16 words: Transformers for image recognition at scale. arXiv.

[58] Chen L.C., Papandreou G., Kokkinos I., Murphy K., Yuille A.L. (2017). Deeplab: Semantic image segmentation with deep convolutional nets, atrous convolution, and fully connected crfs. IEEE Trans. Pattern Anal. Mach. Intell..

[59] Chen L.C. (2017). Rethinking atrous convolution for semantic image segmentation. arXiv.

[60] Chen L.C., Zhu Y., Papandreou G., Schroff F., Adam H. Encoder-decoder with atrous separable convolution for semantic image segmentation. Proceedings of the European Conference on Computer Vision (ECCV).

[61] Yuan Y., Chen X., Wang J. (2020). Object-contextual representations for semantic segmentation. Proceedings of the Computer Vision–ECCV 2020: 16th European Conference.

[62] Zhang H., Zhang H., Wang C., Xie J. Co-occurrent features in semantic segmentation. Proceedings of the IEEE/CVF Conference on Computer Vision and Pattern Recognition.

[63] Huang Z., Wang X., Wang J., Liu W., Wang J. Weakly-supervised semantic segmentation network with deep seeded region growing. Proceedings of the IEEE Conference on Computer Vision and Pattern Recognition.

[64] Kirillov A., Girshick R., He K., Dollár P. Panoptic feature pyramid networks. Proceedings of the IEEE/CVF Conference on Computer Vision and Pattern Recognition.

[65] Xiao T., Liu Y., Zhou B., Jiang Y., Sun J. Unified perceptual parsing for scene understanding. Proceedings of the European Conference on Computer Vision (ECCV).

[66] Zhou Z., Lei Y., Zhang B., Liu L., Liu Y. Zegclip: Towards adapting clip for zero-shot semantic segmentation. Proceedings of the IEEE/CVF Conference on Computer Vision and Pattern Recognition.

[67] He W., Jamonnak S., Gou L., Ren L. Clip-s4: Language-guided self-supervised semantic segmentation. Proceedings of the IEEE/CVF Conference on Computer Vision and Pattern Recognition.

[68] Ma X., Wu Q., Zhao X., Zhang X., Pun M.O., Huang B. (2024). Sam-assisted remote sensing imagery semantic segmentation with object and boundary constraints. IEEE Trans. Geosci. Remote Sens..

[69] Deng J., Dong W., Socher R., Li L.J., Li K., Fei-Fei L. (2009). Imagenet: A large-scale hierarchical image database. Proceedings of the 2009 IEEE Conference on Computer Vision and Pattern Recognition.

[70] Parelius E.J. (2023). A review of deep-learning methods for change detection in multispectral remote sensing images. Remote Sens..

[71] Ronneberger O., Fischer P., Brox T. (2015). U-net: Convolutional networks for biomedical image segmentation. Proceedings of the Medical Image Computing and Computer-Assisted Intervention–MICCAI 2015: 18th International Conference.

[72] LeCun Y., Bengio Y., Hinton G. (2015). Deep learning. Nature.

[73] Glorot X., Bordes A., Bengio Y. Deep sparse rectifier neural networks. Proceedings of the Fourteenth International Conference on Artificial Intelligence and Statistics. JMLR Workshop and Conference Proceedings.

[74] Zhu J., Liao S., Yi D., Lei Z., Li S.Z. (2015). Multi-label cnn based pedestrian attribute learning for soft biometrics. Proceedings of the 2015 International Conference on Biometrics (ICB).

[75] Kingma D.P. (2014). Adam: A method for stochastic optimization. arXiv.

[76] Dong S., Wang L., Du B., Meng X. (2024). ChangeCLIP: Remote sensing change detection with multimodal vision-language representation learning. ISPRS J. Photogramm. Remote Sens..

